# Development of an assessment tool for designated medical institutions in China——Based on the application of an online assessment system

**DOI:** 10.3389/fpubh.2024.1372821

**Published:** 2024-05-06

**Authors:** Qian Wang, Ruiming Dai, Qianqian Yu, Tiantian Zhang, Bin Wu

**Affiliations:** ^1^Fudan Institute on Ageing, Fudan University, Shanghai, China; ^2^Center for Population and Development Policy Studies, Fudan University, Shanghai, China; ^3^School of Public Health, Fudan University, Shanghai, China; ^4^Shanghai Institute of Infectious Disease and Biosecurity, Fudan University, Shanghai, China; ^5^Shanghai Haiyul Information Technology Co. Ltd, Shanghai, China

**Keywords:** basic medical insurance, designated medical institutions, online assessment system, medical insurance supervision, medical service agreement

## Abstract

**Background:**

Due to the expanding coverage of medical insurance and the growth of medical expenses, the ability to assess the performance of designated medical institutions (DMIs) in supporting the delivery of high-quality patient care and the standardized use of funds represents a priority in China. Despite such interest, there has yet to be an operable standard and labor-saving method for assessing DMIs in China.

**Objective:**

The main objectives include two aspects: (1) establishing an evaluation index system for DMIs based on contracts; (2) designing and developing an online evaluation platform.

**Methods:**

A group of 20 experts with theoretical and practical expertise in medical insurance regulation and performance evaluation were invited to select available indicators. A combination weighting method based on analytic hierarchy process and entropy method was used to determine the weight coefficient. Shanghai was taken as the sample area, and 760 DMIs were included in the empirical research. The test-retest reliability method and criterion-related validity method was used to test the reliability and the validity of the evaluation result.

**Results:**

An assessment index system that included 6 domains and 56 indicators was established in this study. Furthermore, we developed an online platform to assist in the implementation of the assessment. The results showed that the average score of assessment was 94.39, the median was 96.92. The test-retest reliability value was 0.96 (P ≤ 0.01), which indicated high stability of the assessment. In addition, there was a significant negative relationship between assessment score and the penalty amount of DMIs (R = −0.133, *P* < 0.001). After adjusting for the basic characteristics of medical institutions, the number of visits and revenue, the negative relationship was still significant (B = −0.080, *P* < 0.05). These results are consistent with expectations, indicating that the assessment had good criterion-related validity.

**Conclusions:**

This study established an operable assessment measure and developed an online platform to assess the performance of DMIs. The results showed good feasibility and reliability in empirical research. Our research findings provided an operable Chinese solution for DMI assessment that saves manpower and time, which would have good enlightening significance in other regions of China and in low-income and middle-income countries internationally.

## Highlights

- An assessment standard based on medical insurance service agreements was established to supervise the performance of contracted medical service providers, and the results showed good feasibility and reliability in empirical results.
- An online platform was developed to assist in the implementation of assessment, which helps save time and manpower in the assessment of medical insurance agencies.

## Introduction

To improve the quality, efficiency, and responsiveness of medical services, market competition mechanisms have been introduced in healthcare industry. In the context of marketization of medical services, contract management is an important management method to improve the performance and accountability of medical institutions in recent years, and has been widely applied in the healthcare field in various countries around the world. In China, the main elements of the basic medical insurance system include medical service providers, medical insurance agencies, medical insurance administrative departments and insured persons (1). Among them, the medical insurance agencies and medical service providers are interrelated through contract. Specifically, the medical insurance agencies play the role of medical service purchasers—they select qualified medical service providers and sign contracts with them, and these contracted medical service providers should provide medical services to the insured persons accordance with the contract requirements. These contracted medical service providers are named designated medical institutions (DMIs) in China. The medical expenses can be partially or fully reimbursed by medical insurance. Therefore, the medical insurance agencies have been exploring how to effectively play the role of contract management and how to strengthen the performance evaluation of DMIs.

Based on the theory of incomplete contracts, the contract between the medical insurance agencies and the medical service provider is a typical incomplete contract, characterized by unpredictability, difficulty in contracting, and difficulty in verification (see Table 1) (2, 3). Due to human's limited rationality and opportunistic behavior, the complexity and uncertainty of the external environment, as well as the asymmetry and imperfection of information, it is impossible to take all possible future scenarios into account when signing the contract, making it difficult for the medical insurance agency to clearly specify the quantity and quality of medical services that DMIs need to provide in contracts, resulting in a lack of effective evaluation criteria for contract execution. In recent years, the default rate of DMIs in China has been high, and the overall implementation of agreements has been poor, seriously endangering the security of medical insurance funds. According to the statistics of the National Healthcare Security Administration, in 2019, a total of 815,000 DMIs were inspected, of which more than 264,000 (approximately 32%) were punished for violating the agreement, and 11.56 billion yuan of medical insurance funds were recovered. In 2020, a total of 627,000 DMIs were inspected, of which 401,000 (64%) were punished, and a total of 22.31 billion yuan was recovered throughout the year (4). Therefore, it is urgent to design effective performance evaluation standards from the perspective of contract management, intuitively reflecting the performance of DMIs, so as to supervise and restrict their behavior and improve the security and efficiency of medical insurance funds.

**Table 1:** Characteristics of contracts between medical insurance agencies and DMIs.

**Characteristics**	**Causes**
Unpredictability	The bounded rationality of contract subjects such as medical insurance agencies and DMIs • The unpredictability of disease, doctor's behavior and medical service effectiveness
Difficulty in contracting	The low measurability of medical service leads to high contracting cost for designing detailed terms
Difficulty in verification	Lack of professional third-party evaluation agency • Lack of operational standards for measuring service outcomes

In an effort to reduce expenditures, improve health outcomes and enhance patient experience, private and public payers have experimented with an array of programs, including hospital report cards, accountable care organizations (ACOs), and pay-for-performance (P4P) programs (5). Hospital report card policies refer to governments publishing quality indicators of hospitals mainly including health outcome indicators such as risk-adjusted mortality rates or readmission rates (6). The ACO model (7) ensures that medical services meet certain quality standards while medical expenses are lower than pre-set cost standards. The more medical expenses saved, the more economic rewards ACO members receive (8–10). In this model, 34 nationally recognized quality measures (four quality domains of patient/caregiver experience, care coordination and patient safety, preventive health and clinical care for at-risk populations) are used to control quality. P4P programs, also known as value-based purchasing (VBP), link provider's reimbursement with performance on a set of defined quality measures (11). For example, the UK National Health Service introduced the Quality of Outcomes Framework (QOF) in 2003, which includes three domains (clinical, public health, quality improvement) of indicators. That same year, the US Centers for Medicare & Medicaid Services (CMS) began the Hospital Quality Incentive Demonstration (HQID), 34 quality measures were established by the CMS across 5 clinical conditions addressing acute care (12). However, the above influential evaluation methods for medical service providers in developed countries are based on solid medical information and professional third-party evaluation institutions (13–17), making it difficult to apply them to low-income and middle-income countries or regions with relatively underdeveloped medical information support facilities and third-party evaluation.

In China, medical insurance payers are increasingly paying attention to the evaluation of medical service providers. In previous studies, research has mainly focused on topics such as performance evaluation, credit evaluation, medical cost detection and identification of abnormal behaviors. For example, a study established a credit evaluation system for public hospitals that included five dimensions—honest procurement, honest charging, honest medical insurance, honest diagnosis and treatment, and honest practice, and tested it in three tertiary medical institutions (18). Another study improved clustering-based local outlier factor methods to detect abnormal medical fees and utilized rule-based methods to identify abnormal medical behavior, such as hospitalization decomposition (19). These studies provide valuable contributions to the supervision and decision-making of medical insurance payers. However, the existing literature on the performance evaluation of contracted medical service providers is mostly limited to specific aspects such as medical quality, medical behavior, and medical expenses, and there is a lack of comprehensive consideration based on contracts. Besides, most studies were conducted mainly in public tertiary hospitals with complete medical information infrastructure, resulting in limited generalizability of the evaluation indicators. The other types of medical service providers may be difficult to implement due to operational difficulties such as data collection and insufficient on-site assessment manpower (20). Therefore, this study aims to construct an evaluation index system to identify the compliance level of DMIs, providing reference for medical insurance supervision. Based on this, an online evaluation platform was designed and developed to collect, calculate, and analyze evaluation data, solving the problem of insufficient manpower in medical insurance contract management.

## Methods

### Participants

**Figure 1:**
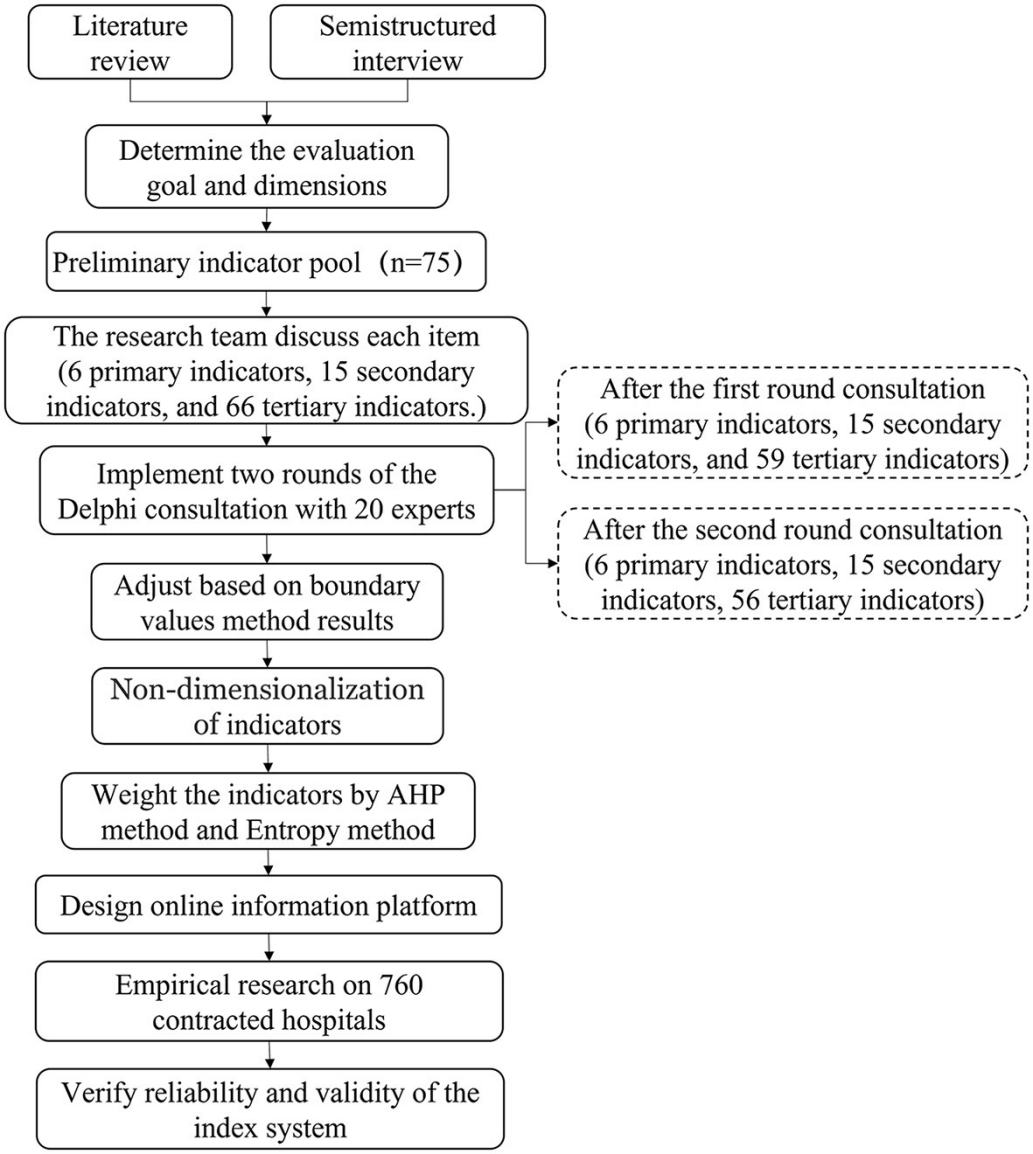
Study procedure for constructing an evaluation index system of DMIs.

The procedure of this study is shown in Figure 1. Firstly, we established an evaluation index system for DMIs based on contracts. Secondly, we designed and developed an online platform to implement the evaluation. Thirdly, we conducted empirical research on 760 DMIs and tested the reliability and validity of the indicator system.

In the empirical research, we took Shanghai as the sample area. The inclusion criteria for the evaluated institutions were as follows: (1) should remain in business by December 31, 2020; (2) should be the headquarters of the contracted institutions. The exclusion criteria were as follows: (1) internal medical institutions affiliated with enterprises, schools, and other institutions, which do not provide health services to the public; (2) branch medical institutions, such as the branch hospital of a public medical institution. According to the above inclusion and exclusion criteria, 760 contracted health providers in Shanghai were included in the study.

### Determine the evaluation goal and dimensions

Based on contract theory and performance management theory, we analyzed the elements of the contract-based medical service providers evaluation system and the logical relationships between them through literature review and expert interviews. Then we constructed a conceptual framework (see Appendix 1) including evaluation subject, evaluation object, evaluation goal, evaluation dimensions, and evaluation tool for evaluation. Specifically, the evaluation subjects refer to medical insurance agencies, the evaluation objects refer to DMIs and the evaluation tool refer to online platform. The determination of evaluation goals and evaluation dimensions was as follows:

Firstly, we systematically collected policy documents published from 2009 to 2020 on official platforms such as the Chinese government website, the National Health Commission, and the National Healthcare Security Administration. A total of 23 national policy documents and 114 local policy documents were collected. After sorting and analyzing, we summarized the development stages and goals of basic medical insurance and contract management in China, and analyzed the main goals of contract management at the current stage.

At the same time, semistructured interviews with 12 experts (consisting of five medical insurance administrators, four hospital managers and three scholars) were conducted with the following questions being asked: “What do you think are the goals of contract-based assessment for designated medical institutions?” and “What requirements do you think designated medical institutions should meet?”

Based on the results of the above two parts, we have determined the goals and dimensions for evaluating the compliance level of DMIs.

### Building the preliminary indicator pool

A literature review of on the performance of contracted medical service providers was performed by searching PubMed, CNKI and Wanfang databases to collect preliminary evaluation indicators. We collected literatures from 2000 to 2021, and gathered a total of 660 articles. After excluding the duplicates, conference reports, and papers without any indicators, 120 papers were left. Besides, we also included 33 local medical insurance contracts from different regions for in-depth review together. After reviewing the above materials, a total of 75 indicators were identified. The research team discussed each item one by one, deleted the duplicate ones, and classified them to form a hierarchical index system consisting of six primary indicators, 15 secondary indicators, and 66 tertiary indicators.

### Using the Delphi method to build an index system

Two rounds of the Delphi consultation were conducted to collect experts' opinions on the preliminary index system. We selected 20 experts including medical insurance administrators, hospital managers and scholars to participate in the Delphi consultation, and invited them to score the importance and feasibility of each candidate indicator on a 1–10 scale.

And then, we used the boundary values of two important statistics (arithmetic mean and coefficient) to screen the indicators. The inclusion criteria were as follows: (1) the arithmetic mean of the importance and feasibility of candidate indicators is >7; (2) the coefficient of variation is ≤ 0.25. Otherwise, the indicators will be deleted.

To ensure the scientific soundness and rationality of the Delphi method, the experts' positive coefficient, authority coefficient and coordination coefficient were calculated. The experts' positive coefficient reflects the positive input from the experts, which can be expressed by the effective response rate. An effective response rate above 70 is considered a very good standard. The expert authority coefficient is generally determined by two factors: the judgment basis coefficient, denoted by Ca, and the familiarity coefficient, denoted by Cs. The calculation formula for expert authority coefficient is $C_{r}=\frac{C_{a}+C_{s}}{2}$. Generally, the higher Cr is, the higher the prediction accuracy. A Cr value < 0.7 is considered to indicate acceptable reliability. The coordination of the experts' opinions reflects the consistency of evaluation of all experts, which guarantees the scientific of the index system, and can be calculated by Kendall's W concordance coefficient.

### Exploring the quantitative calculation method

#### Dimensionless methods

Dimensionless methods were explored based on the data type to obtain the standard value of each indicator. The assignment methods for standard values in this study were as follows (21–24): (1) “0–1” assignment method, which is applicable to binary classification indicators with the answer of “yes” or “no”. (2) Multi-condition assignment method, which is applicable to indicators that need to meet more than one requirement. (3) Proportional assignment method, which is applicable to indicators expressed as a percentage. (4) Segment assignment method, which is applicable to indicators with an acceptable range, with a value of 0 assigned outside the acceptable range and a proportional value assigned within the acceptable range. (5) Min-max normalization, which is applicable to indicators lacking reference values and without extreme outliers. The calculation formula is as follows when the indicator is positive in direction (which means the higher the indicator value is, the better the evaluation will be):


$$\begin{array}{l} y_{i}=\frac{x_{i}-\min\left(X_{i}\right)}{\max\left(x_{i}\right)-\min\left(x_{i}\right)} \end{array}$$

The calculation formula is as follows when the indicator is negative (which means the lower the indicator value is, the worse the evaluation will be):


$$\begin{array}{l} y_{i}=\frac{\max\left(x_{i}\right)-x_{i}}{\max\left(x_{i}\right)-\min\left(x_{i}\right)} \end{array}$$

Where *x*_*i*_ is the original value of the indicator, *y*_*i*_ is the standard value of the indicator.

(6) Horizontal comparison assignment, which assigns values by comparing the scores of the evaluated institution with the mean of all assessed objects, is used for indicators lacking reference values and with extreme outliers. The calculation formula is as follows when the indicator is positive in direction:


$$\begin{array}{l} y_{i}=\frac{X_{i}}{\overset{\bar{X}}{\;}} \end{array}$$

The calculation formula is as follows when the indicator is negative direction:


$$\begin{array}{l} y_{i}=\frac{\overset{\bar{X}}{\;}}{X_{i}} \end{array}$$

Where *x*_*i*_ is the original value of the indicator, *y*_*i*_ is the standard value of the indicator, $\overset{\bar{\;}}{X}\;$is the average value of similar medical institutions at the same level.

#### Combination weighting method

A combination weighting method based on analytic hierarchy process (AHP) method and entropy method was used to determine the weight coefficient.

##### Using the AHP to assign subjective weights

AHP method was used to determine the subjective weight coefficient. Twenty experts who participated in consultation compared the relative importance of the indicators in each domain, scored them and formed a judgment matrix A = (_*a*_*ij*_)*n*×*n*_ (25). Yaahp11.2 was used to input the judgment matrix of each expert and calculate the subjective weight coefficient of each indicator.

##### Using the entropy method to assign objective weights

Entropy method was used to determine the objective weight coefficient, and the calculation formula was as follows (26).


(1) $$\begin{array}{l} P_{ij}=\frac{x_{ij}}{\overset{m}{\underset{i=1}{\sum }}x_{ij}} \end{array}$$

*P*_*ij*_ means the proportion of the index value of medical institution i of index j.


(2) $$\begin{array}{l} e_{j}=-k\overset{m}{\underset{i=1}{\sum }}P_{ij}\ln\;P_{ij} \end{array}$$

*e*_*j*_ means the entropy of index j.


(3) $$\begin{array}{l} {g_{j}=1-e}_{j},\left(j=1,2,3\ldots n\right) \end{array}$$

*g*_*j*_ means the difference coefficient of index j.


(4) $$\begin{array}{l} \mu_{j}=\frac{g_{j}}{\overset{n}{\underset{j=1}{\sum }}g_{j}}\left(j=1,2,\ldots ,n\right) \end{array}$$

μ_*j*_ means correction coefficient of index j.


(5) $$\begin{array}{l} \theta_{j}=\frac{\mu_{j}w_{j}}{\overset{n}{\underset{j=1}{\sum }}\mu_{j}w_{j}} \end{array}$$

*w*_*j*_ means the initial weight obtained by AHP method, θ_*j*_ means the weight obtained by adjusting *w*_*j*_ with the correction coefficient μ_*j*_.

The combination weight W is calculated by the initial weight (*w*_*j*_) obtained by AHP method and the adjusting weight (θ_*j*_) obtained by entropy method. The formula is as follows, and the value of ρ is usually 0.5.


(6) $$\begin{array}{l} W_{j}=\rho w_{j}+\left(1-\rho \right)\theta_{j} \end{array}$$

*W*_*j*_ means the combination weight calculated by *w*_*j*_ and θ_*j*_.

### Reliability and validity analysis

#### Reliability analysis

We used the test-retest reliability method to test the reliability of the indicator system. The correlation coefficient of the assessment scores obtained online and on site was calculated as the retest reliability value to test the stability of the assessment.

#### Validity analysis

We use criterion-related validity to test the consistency between the measured value and the true value of the evaluation result. The criterion is usually represented by a recognized, reliable and authoritative indicator. The higher the correlation between evaluation results and the criterion, the more authentic and reliable the evaluation results are. In this study, the criterion indicator is represented by the penalty amount for medical insurance supervision of DMIs. The research hypothesis is that when there is a significant negative correlation between the evaluation value and the criterion value (which means the higher the evaluation score, the lower the penalty amount of contracted medical providers), it indicates that the evaluation result is consistent with the true value, and the assessment result is reliable. We used spearman correlation analysis and linear regression analysis to analyze the relationship between evaluation results and the criterion indicator.

### Design of the online assessment platform

The online assessment platform mainly aims at solving the following problems: (1) the number of DMIs to be assessed is large, and the manpower for assessment is insufficient; (2) the assessment data comes from multiple departments and 760 DMIs, which means that the data sources are diverse and the data volume is large. Thus, it is difficult to integrate, process and analyze the data; (3) different levels and types of medical institutions would be assessed by different indicators, which makes manual data collection and calculation prone to errors.

**Figure 2:**
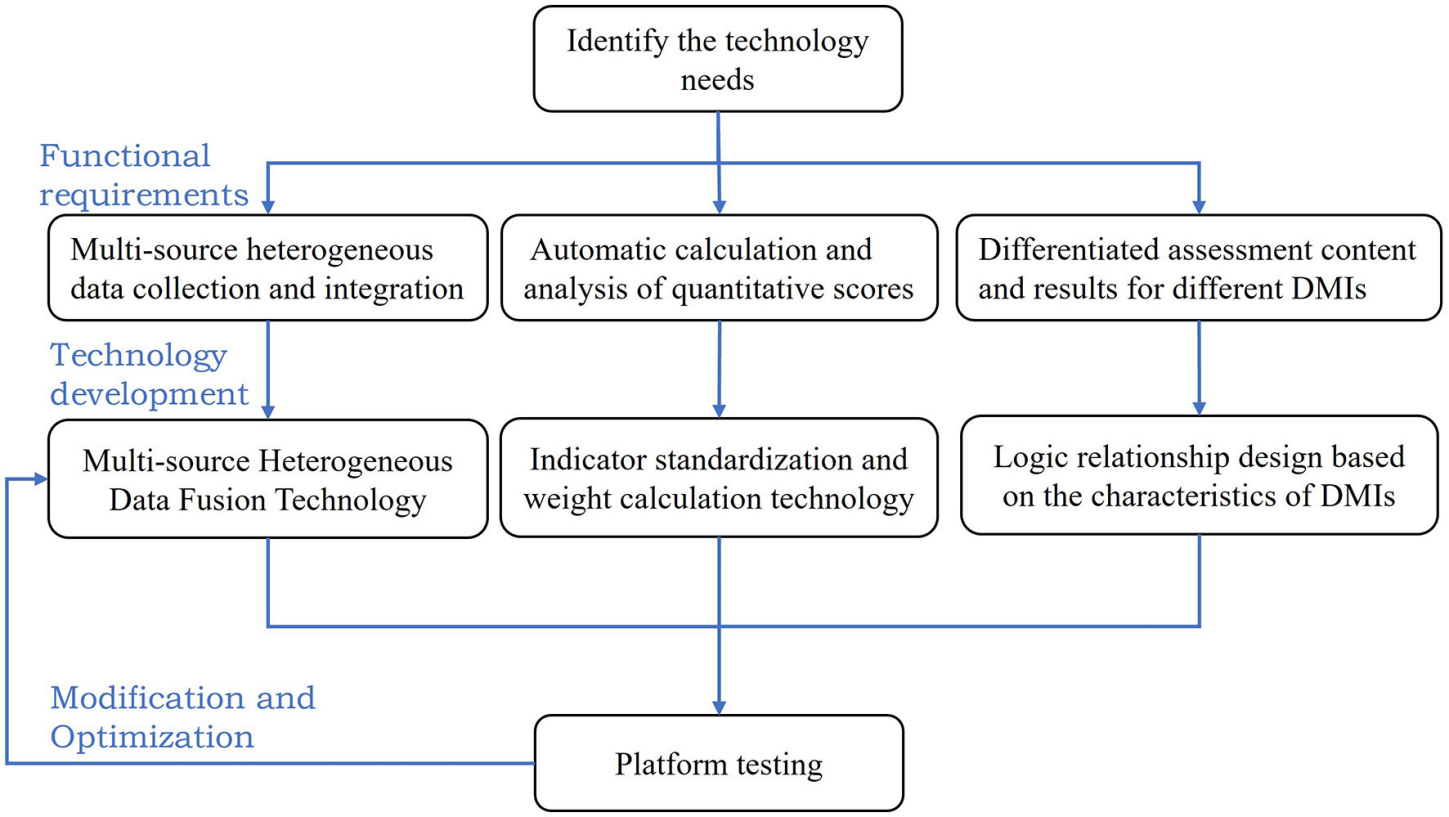
Technical development of the assessment platform for DMIs.

Based on the above problems, the design of the information platform was as follows: (1) Automatic calculation and analysis of quantitative scores. Based on the indicator standardization and weight calculation method obtained from the above research, a score calculation program was designed to automatically calculate the assessment scores. (2) Technology for the collection and integration of multisource heterogeneous data. As the data came from multiple sources, the research group developed the integration and standardized technology of multisource heterogeneous data for the assessment platform. (3) The logical correlation design based on the characteristics of medical institutions. Due to the differences in the medical insurance settlement level, nature and type of evaluated institutions, the platform established a logical association between the assessment content, assessment results and characteristics of medical institutions, which made it possible to implement differentiated assessments for different medical institutions (see Figure 2).

### Statistical analysis

The arithmetic mean, standard deviation and coefficient of each indicator's importance and feasibility scores were used to screen the indicators. The experts' positive coefficient, authority coefficient and the Kendall W coefficient were used to determine whether the Delphi expert consultation results were scientific and reliable. An analytic hierarchy process (AHP) method and entropy method was used to determine the weight coefficient. The Cronbach's α reliability coefficient method and the criterion-related validity were used to test the reliability and validity of evaluation. All the above analysis were conducted in SPSS 27.0, Excel 2021 or Yaahp11.2. A two-sided p-value < 0.05 was considered statistically significant in this study.

## Results

### Definition of assessment objectives and dimensions

According to the policy document review and interviews results, the compliance level of DMIs was defined as the performance evaluation of medical service providers conducted by medical insurance management departments or third-party organizations based on contracts. The aim is to use limited health insurance funds to purchase better quality services, and improve the medical experience of insured persons. Thus, the assessment objectives mainly include three aspects: standard use of medical insurance funds, providing high-quality, efficient and affordable medical services, and improving the medical experience of insured persons. On this basis, six assessment dimensions are further formed, including medical insurance management, medical insurance settlement, medical service quality, medical service efficiency, medical expense control, and experience of the insured persons. The specific definitions of each assessment dimension are shown in Table 2.

**Table 2:** Six main domains and connotations of the assessment index system.

**Domains**	**Connotation**
Medical insurance management	Refers to the infrastructure configuration and completion of basic business required for medical insurance management
Medical insurance settlement	Refers to the management and settlement of medical insurance funds by DMIs comply with regulations
Medical service quality	Refers to the medical service quality and safety of DMIs
Medical service efficiency	Refers to the convenience and efficiency of diagnosis and treatment provided by DMIs
Medical expense control	Including the growth of medical expenses and the rationality of medical fees
Experience of the insured persons	Refers to the protection of the rights of the insured and the subjective experiences of the insured persons

### Results of the Delphi consultation

#### Basic information of experts

Among the experts participated in the Delphi consultation, the majority had a master's degree or above (95%), 80% had been engaged in relevant work for more than 10 years, and 85% of them held senior professional title. Thus, they had authoritative opinions on management of medical service providers. More details are shown in Table 3.

**Table 3:** Basic information of experts (*N* = 20).

**Participants' information**	***N***	**%**
**Gender**		
Male	13	65.0
Female	7	35.0
**Age**		
30–39	6	30.0
40–49	8	40.0
≥50	6	30.0
**Education**		
Bachelor	1	5.0
Master	12	60.0
PhD	7	35.0
**Occupation**		
Hospital manager	5	25.0
Medical insurance administrators	6	30.0
Health department administrators	3	15.0
Scholar	6	30.0
**Professional title**		
Senior	17	85.0
Middle	3	15.0
**working years**		
0–9	4	20.0
10–19	5	25.0
20–29	5	25.0
≥30	6	30.0

#### Experts' positive coefficient

In the first round of the Delphi consultation, all 20 questionnaires were recovered, with a response rate of 100%. In the second round, 20 questionnaires were distributed and 19 were collected back, with a response rate of 95.00%. The response rate of all the two rounds of the Delphi consultations were both above 70%, indicating that the experts' feedback was positive.

#### Expert authority coefficient (Cr)

The values of the expert authority coefficient Cr from the two rounds of expert consultations were 0.85 and 0.86 respectively, >0.7, indicating that the expert consultation results were accurate and reliable.

#### Coordination of experts' opinions

The coordination of experts' opinions is shown in Table 4. In both two rounds of the Delphi consultations, Kendall's W coefficients ranged between 0.150 and 0.426, and the importance and feasibility scores in both rounds were all effective (*P* < 0.001), suggesting the consistency among experts.

**Table 4:** Kendall's W concordance coefficient test results.

	**First round**		**Second round**	
	**Importance**	**Feasibility**	**Importance**	**Feasibility**
Kw	0.308	0.150	0.426	0.153
χ^2^	633.480	306.811	853.746	312.418
*P*	< 0.001	< 0.001	< 0.001	< 0.001

#### Indicators' screening

We calculated the boundary values of all indicators. According to the screening criteria, the primary and secondary indicators met the retention criteria, and the tertiary indicators were adjusted after the consultation. Seven indicators were deleted in the first round, and three were deleted in the second round (see Appendix 2).

The adjusted assessment index system for DMIs included 6 domains and 56 indicators. The 56 indicators and their descriptions, data sources, and directions were shown in Appendix 3. The data sources for the assessment mainly included the following: (1) hospital self-reported data, which was collected from the assessed DMI reporting the required data and uploading supporting materials on the information platform; (2) Data records from the relevant administrative department, which were collected from the relevant administrative department supplying daily work data and previous inspection data related to the assessed DMI.

### Results of the quantitative calculation method

#### Dimensionless results

Based on the above criteria, in this study, the dimensionless method and assignment criterion for each indicator were chosen according to the data type, and the standard values were calculated. The specific results are shown in Appendix 4.

#### Results of the combination weight coefficient

Table 5 shows the combination weighting coefficients calculated by the AHP and entropy methods. The weight of the six domains of agreement enforcement assessment were ranked from high to low as follows: medical service quality, medical expense, medical service efficiency, medical insurance settlement, experience of the insured, and medical insurance management. Improving the quality of medical services and reducing expenditures are the most important aspects of performance evaluation for DMIs.

**Table 5:** Weight coefficients of indicators.

**Indicators**	** $w_{j}^{\text{a}}$ **	** $\theta_{j}^{\text{b}}$ **	** $W_{j}^{\text{c}}$ **
**1. Medical insurance management**	0.0748	0.0295	0.0521
**1.1 Basic construction**	0.0133	0.0013	0.0073
1.1.1 Establish medical insurance department	0.0105	0.0009	0.0057
1.1.2 Build bylaws and policies	0.0028	0.0004	0.0016
**1.2 Human resource management**	0.0066	0.0028	0.0047
1.2.1 Records management of physicians	0.0019	0.0019	0.0019
1.2.2 Records accuracy of physicians	0.0007	0.0007	0.0007
1.2.3 Insurance settlement personnel	0.0040	0.0002	0.0021
**1.3 Information system**	0.0247	0.0041	0.0144
1.3.1 Establish medical insurance information management department	0.0050	0.0002	0.0026
1.3.2 Connect medical insurance network	0.0093	0.0005	0.0049
1.3.3 Equip auxiliary equipment in computer room	0.0012	0.0001	0.0006
1.3.4 Equip intelligent monitoring system of basic medical insurance	0.0014	0.0014	0.0014
1.3.5 Establish doctor (nursing) workstation	0.0020	0.0011	0.0016
1.3.6 Internet security	0.0047	0.0007	0.0027
1.3.7 Contingency plan for information system	0.0012	0.0001	0.0006
**1.4 Medical insurance business**	0.0262	0.0148	0.0205
1.4.1 Sign of designated medical institution	0.0021	0.0010	0.0015
1.4.2 Monitoring equipment in medical insurance service area	0.0013	0.0006	0.0010
1.4.3 Medical insurance policy consulting service	0.0031	0.0031	0.0031
1.4.4 Medical insurance policy training for medical personnel	0.0043	0.0009	0.0026
1.4.5 Publicity of medical insurance complaint channels	0.0043	0.0002	0.0023
1.4.10 Implementation of additional agreements of specific institutions	0.0111	0.0090	0.0100
**1.5 Drug procurement**	0.0038	0.0065	0.0052
1.5.1 Purchase, sales and deposit record	0.0007	0.0001	0.0004
1.5.2 Application of national procurement platform	0.0020	0.0020	0.0020
1.5.4 Completely product authorization information	0.0003	0.0003	0.0003
1.5.5 Proportion of centralized procurement drugs	0.0008	0.0042	0.0025
**2. Medical insurance settlement**	0.1088	0.139	0.1241
**2.1 Claims settlement requirement**	0.0347	0.0223	0.0285
2.1.1 Claims settlement materials	0.0145	0.0051	0.0098
2.1.2 Scope of claim settlement	0.0089	0.0089	0.0089
2.1.3 Issue settlement bills	0.0032	0.0002	0.0017
2.1.4 Settlement of agreed diagnosis and treatment items	0.0081	0.0081	0.0081
**2.2 Reconciliation management**	0.0744	0.1167	0.0956
2.2.1 Overdue days of reconciliation	0.0189	0.0016	0.0103
2.2.2 Proportion of daily reconciliation deduction amount	0.0555	0.1151	0.0853
**3. Medical service quality**	0.4004	0.4471	0.4272
**3.1 Medical service management**	0.1110	0.1142	0.1127
3.1.1 Identify the insured correctly	0.0144	0.0144	0.0144
3.1.2 Qualified medical record	0.0167	0.0050	0.0109
3.1.3 Medical expense inquiry service	0.0102	0.0102	0.0102
3.1.4 Registration and filing of external inspection and treatment	0.0044	0.0002	0.0023
3.1.5 Standard use of family sickbeds	0.0079	0.0079	0.0079
3.1.8 Outpatient prescription outsourcing service	0.0043	0.0043	0.0043
3.1.11 Hospitals reject patients without justifiable reasons	0.0322	0.0322	0.0322
3.1.12 Scoring of bad practice of medical institutions	0.0210	0.0400	0.0305
**3.2 Health care quality management**	0.2961	0.3329	0.3145
3.2.1 Qualified rate of inspection	0.0526	0.0185	0.0356
3.2.2 Proportion of default amount of drugs with payment limitation	0.1880	0.2647	0.2263
3.2.4 Mortality of cases in low-risk group	0.0555	0.0497	0.0526
**4. Medical service efficiency**	0.1095	0.0761	0.0896
**4.1 Convenient medical treatment**	0.0261	0.0153	0.0207
4.1.1 Average waiting time after appointment	0.0199	0.0150	0.0175
4.1.2 Convenience Services and Facilities	0.0062	0.0003	0.0032
**4.2 Efficient diagnosis and treatment**	0.0771	0.0608	0.0689
4.2.1 Outpatient return visit rate	0.0171	0.0178	0.0174
4.2.2 Re admission rate within 15 days after discharge	0.0449	0.0242	0.0346
4.2.5 Inpatient outpatient ratio	0.0151	0.0188	0.0169
**5. Medical expense**	0.2306	0.2797	0.2569
**5.1 Growth rate of medical expenses**	0.0931	0.1817	0.1374
5.1.1 Proportion of medical service income	0.0289	0.1037	0.0663
5.1.2 Increase in average outpatient cost per time	0.0227	0.0371	0.0299
5.1.3 Increase in average hospitalization cost per time	0.0232	0.0212	0.0222
5.1.4 Increase in average drug cost per outpatient	0.0083	0.0040	0.0061
5.1.5 Increase in average drug cost per hospitalization	0.0100	0.0157	0.0129
**5.2 Reasonable medical charges**	0.1411	0.098	0.1195
5.2.5 Cost shifting of exceeding medical insurance settlement	0.0525	0.0132	0.0328
5.2.6 Implementation of copay rate of medical insurance	0.0748	0.0710	0.0729
5.2.9 Standardizing charge for newly increased medical service	0.0138	0.0138	0.0138
**6. Experience of the insured**	0.0760	0.0286	0.0503
**6.1 The insured's rights**	0.0294	0.0193	0.0244
6.1.1 Signing of informed consent	0.0198	0.0120	0.0159
6.1.2 Information security	0.0096	0.0073	0.0085
**6.2 Evaluation of the insured**	0.0425	0.0093	0.0259
6.2.1 Subjective satisfaction of the insured	0.0167	0.0030	0.0098
6.2.2 Complaints of the insured	0.0258	0.0063	0.0161

${\;}^{\text{a}}w_{j}$, means subjective weight obtained by AHP method.

${\;}^{\text{b}}\theta_{j}\;$means objective weight obtained by entropy method.

${\;}^{\text{c}}W_{j}$ means combined weight obtained by AHP method and entropy method.

### Construction of the online assessment platform

**Figure 3:**
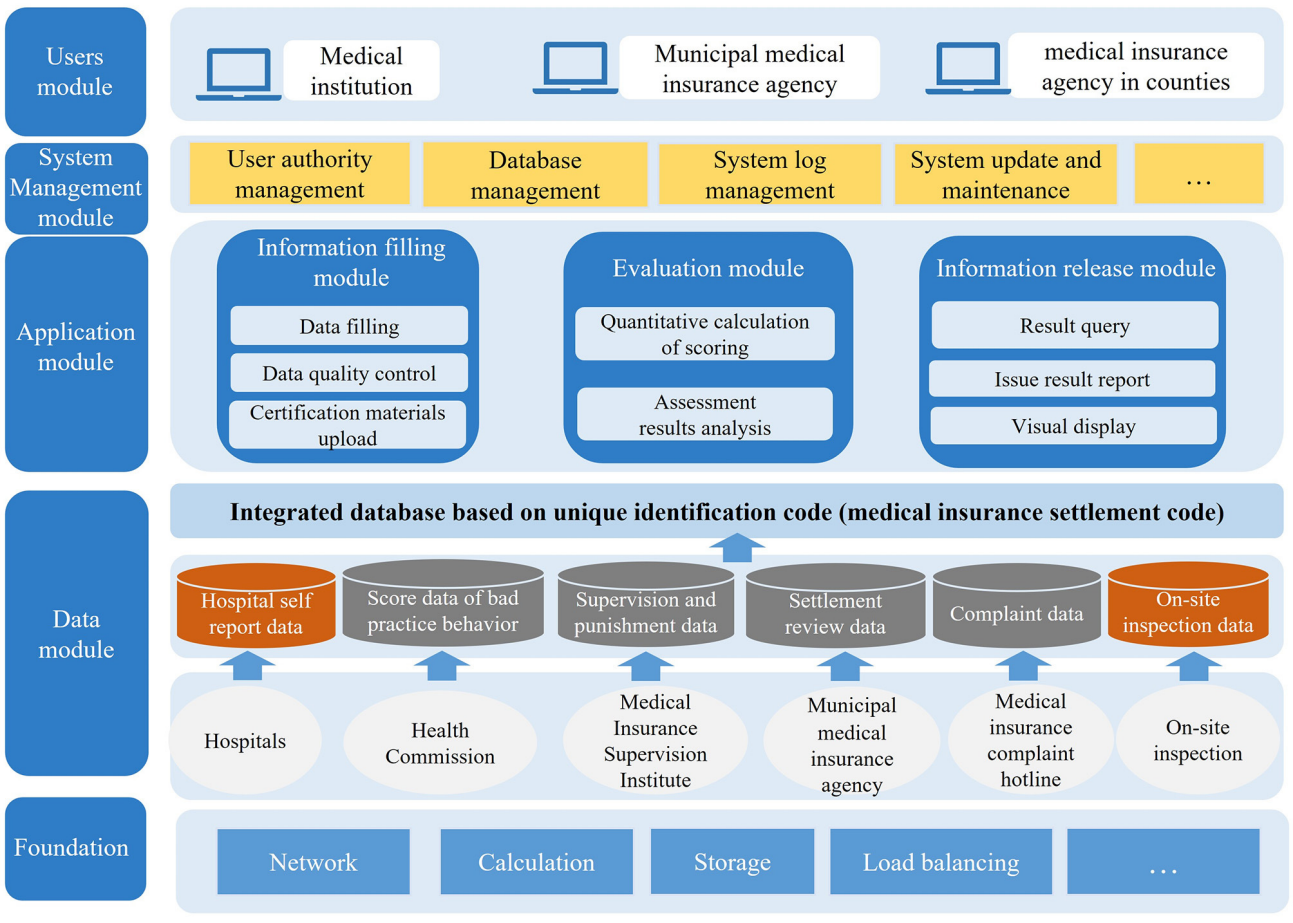
The structure and functions of the assessment information platform.

The overall structure and functions of the online information platform are shown in Figure 3.

The main user groups of the platform include municipal level medical insurance management department, district-level medical insurance management department and contracted medical service institutions. The functions of the platform designed for each user group were as follows: (1) for municipal level medical insurance management department, the main functions of platform include uploading the relative regulatory inspection and work record data, collecting assessment data from medical institutions, automatically calculating the assessment scores, and analyzing, ranking and publishing the assessment results; (2) for district-level medical insurance management department, the main functions of platform include supervising the progress of the assessment, uploading the on-site spot checks data and analyzing the assessment results within the jurisdiction; (3) for contracted medical service institutions, the main functions of platform include uploading the required data of assessment online and querying the assessment results. Therefore, the platform designed the following functional modules: (1) information filling module; (3) scoring module; (4) information release module. Based on the above design, the platform was used to achieve convenient and efficient data collection and integration, automate data calculation and analysis, and visualize assessment results to improve the operability of the assessment.

### Results of empirical research

The arithmetic mean of the assessment score was 94.39, the median was 96.92, the highest score was 100, and the lowest was 60.64.

The study analyzed the assessment scores of contracted medical service providers with different administrative levels, natures and types, and found significant differences in assessment scores between evaluated institutions with different administrative levels (*F* = 45.233, *P* 0.001) (Table 6).

**Table 6:** The assessment scores of DMI.

**Variables**	**χ±σ**	$\frac{t}{F}$	***P***
**Administrative level**			
First-level and below	95.43 ± 6.15	**45.233**	**< 0.001**
Second-level	93.37 ± 6.54		
Third-level	87.15 ± 10.26		
Nature of ownership			
Social-run institutions	94.43 ± 7.12	0.108	0.982
Public institutions	94.37 ± 6.96		
Medical institution type			
comprehensive institutions	94.03 ± 7.40	5.205	0.082
Specialized institutions	94.94 ± 6.37		

The bold values means statistically significant.

### Validation of the assessment score

#### Reliability test

The study ensured the stability and repeatability of the assessment results through the following methods: (1) the interference of manual calculation errors was eliminated through an automatic calculation program, which ensured the accuracy of the data calculation. (2) using the method of complete random sampling, 145 institutions were selected from all evaluated institutions, and data was collected on-site again, and the evaluation results were calculated by experts. The correlation coefficient of the assessment scores obtained online and on site was calculated as the retest reliability value to test the stability of the assessment. The test-retest reliability value was 0.96 (*P* ≤ 0.01), which indicated high stability of the assessment.

#### Results for criterion-related validity

The relationship between the penalty amount and the assessment score of DMIs are shown in Table 7. The correlation analysis result shows that there is a significant negative relationship between the assessment score and the penalty amount of DMIs (R = −0.133, *P* < 0.001). This result is consistent with the hypothesis, and it can be considered that the evaluation results can better reflect the real situation with high validity.

**Table 7:** Correlation analysis between assessment scores and penalty amount of DMIs.

**Variables**	**Assessment scores**	**Penalty amount of DMIs**
Assessment scores	1	−0.133^***^
Penalty amount of designated medical institutions	-	1

^***^P < 0.001.

Inn regression analysis, we adjusted administrative level, medical institution type, the number of medical visits and revenue, and found that the relationship between the assessment score and the penalty amount of DMIs was still significant (*P* < 0.001). Besides, we analyzed the relationship between the assessment score and the penalty amount of DMIs in different levels of medical institutions, and found that the relationship between the assessment score and the penalty amount of DMIs was significant in first-level and third-level medical institutions (*P* < 0.001), see Table 8.

**Table 8:** Linear regression results between assessment scores and penalty amount of DMIs.

**Variables**	**Administrative level**							
	**All DMIs**		**First level and below**		**Second level**		**Third level**	
	**Beta**	**T**	**Beta**	**T**	**Beta**	**T**	**Beta**	**T**
Administrative level	−0.153^***^	−3.455						
Medical institution type	0.039	1.127	0.018	0.353	0.002	0.021	0.147	1.198
Number of Outpatients	−0.127	−1.838	−0.005	−0.106	−0.240^*^	−2.216	−0.120	−0.574
Number of inpatients	−0.213^***^	−4.832	−0.076	−1.823	−0.097	−0.933	−0.140	−0.678
Outpatient Revenue	−0.322^**^	−3.117	0.086	1.828	0.140	1.611	−0.293^*^	−2.470
Inpatient Revenue	0.249^*^	2.413	−0.229^***^	−4.885	0.153	1.736	−0.297	−2.522
Penalty amount of contracted medical providers	**−0.086** ^*^	**−2.452**	**−0.087** ^*^	**−2.037**	0.005	0.058	**−0.236** ^*^	**−2.013**
R^2^	0.143		0.056		0.091		0.255	

^*^P < 0.05, ^**^P < 0.01, ^***^P < 0.001. The bold values means statistically significant.

## Discussion

The main contributions of this study include two aspects: (1) established a unified and operable assessment standard for medical insurance agencies to assess the performance of DMIs; (2) designed and developed an online assessment platform to assist in data collection, submission of supporting materials, score calculation, results analysis and results reporting, which help to improve the efficiency of assessment and save manpower.

In 2020, the coverage of medical insurance reached 95% in China, including over 1.36 billion people (27). Basic medical insurance is highly important for residents' health. Due to the expanding coverage of medical insurance and the growth of medical expenses, the ability to assess the performance of DMIs in supporting the delivery of high-quality patient care and the standardized use of funds represents a priority for all health care systems. In China, such interests are growing. However, the lack of standards and manpower makes it challenging to assess the medical service providers. Most related studies focused on medical insurance payment reform (28–31), credit evaluation of DMIs (32, 33), medical insurance fraud behavior (34, 35) and high medical expense warnings (36, 37). However, most of them reported limitations of data collection (37) and sample's representativeness (32).

In this study, we established an online assessment platform that combined online data reporting with integrated data from relevant administrative departments. Through this platform, we have addressed the data collection issues caused by incomplete medical information infrastructure and insufficient regulatory manpower. First, the construction of information system made it possible to collect data more efficiently. The data of DMIs and relevant administrative departments could be collected through self-reporting and uploading required data records and attachment materials, avoiding the high manpower and low efficiency issues of traditional on-site inspection, which made it possible to assess all the 760 DMIs in Shanghai. Second, the online assessment system integrated data from different departments to ensure the comprehensiveness of data collection. The data collection includes the hospitals' self-report data, hospitals' supportive materials, relevant government administrative departments data (the municipal health commission, health supervision institute, food and drug administration and so on) and daily work records of medical insurance agency.

Based on analysis of the agreement terms, we constructed an index system that includes six dimensions: medical service quality, medical expense, medical service efficiency, medical insurance settlement, experience of the insured, and medical insurance management. The selection of the above dimensions combined the opinions of different stakeholders, in order to reach consensus on performance balance from different perspectives. The Delphi method was used to screen indicators, which had also been well applied in other related studies in China. In addition, in order to prevent possible intentional concealment or non-reporting by hospitals, this study adopted various methods to ensure data quality: (1) intelligent identification of abnormal data in hospital filled out data; (2) Self reporting data needs to be synchronously uploaded with supporting materials for verification; (3) Spot check the evaluated institution and verify the evaluation results through on-site inspection by experts. The empirical results indicate that the assessment tool developed in this study has high feasibility and reliability. In future research, the evaluation platform will be further developed as a workstation for medical insurance agencies to collect daily work records of hospitals, in order to achieve scheduled tracking and dynamic management of DMI.

The main significance of performance assessment is to identify the problems existing in contracted medical service providers. Based on the assessment results, how to motivate medical service providers to continuously improve quality is the next step that needs to be addressed when applying the research outcomes to practice. We suggest that further research and policy development are needed to build based on our research. For example, in empirical research in Shanghai, we attempted to use the assessment results for decision-making by medical insurance agencies as a basis for renewing their contracts with DMIs. A qualification line was set up according to the analysis of assessment scores. Only those qualified institutions could renew agreements with medical insurance agency, and those unqualified ones should suspend the renewal until they were qualified. In future research, more applications based on assessment should be designed, such as grading the institutions and associating the medical insurance payment proportion and inspection frequency with the grade.

### Limitations

The main limitations of this study were as follows. First, the medical service agreement between DMIs and medical insurance agencies may change with the adjustment of medical insurance policies, so in future applications, it is necessary to fine tune the indicators according to policy changes. Second, we selected Shanghai as the sample region for this study. There may be differences in medical insurance requirements among different regions, which may limit the extrapolation of research results. We compared and analyzed service agreements in different regions and found that due to the consistency of medical insurance regulatory goals, the agreement terms in most regions are highly consistent, and only a few terms may have differences. Therefore, our research findings still have good enlightening significance in other regions of China and in low-income and middle-income countries internationally. In subsequent research, the online platform will be further optimized to assist in establishing a continuous quality improvement mechanism for DMIs. All evaluation results of DMI in the past will be stored in the database of the information system, and medical insurance institutions can monitor the quality improvement of DMI through regular inspections.

### Conclusions

The reform of China's medical insurance system has entered a new stage after more than 20 years of practice, which has brought with increasing requirements on the high-quality patient care and the standardized use of funds provided by DMIs. Based on the agreements assigned by medical insurance agency and DMIs, this study established an operable assessment measure and developed an online platform to assess the enforcement of medical service agreements of DMIs. The empirical results of Shanghai indicated that our assessment measures performed well in feasibility and reliability. Besides, the development of online platform improved the efficiency and convenience of assessment, which provided an operable solution to save time and manpower in the supervision of DMIs.

## Data availability statement

The raw data supporting the conclusions of this article will be made available by the authors, without undue reservation.

## Author contributions

QW: Conceptualization, Funding acquisition, Methodology, Project administration, Writing – original draft, Writing – review & editing. RD: Methodology, Validation, Visualization, Writing – review & editing. QY: Writing – review & editing. TZ: Conceptualization, Funding acquisition, Project administration, Resources, Visualization, Writing – review & editing. BW: Software, Visualization, Writing – review & editing.
