# Supplementary material for: Development of an assessment tool for designated medical institutions in China——Based on the application of an online assessment system
Source: Front Public Health. 2024 May 6;12:1372821. doi: 10.3389/fpubh.2024.1372821 (PMC11102995; doi:10.3389/fpubh.2024.1372821)

**Appendix 1**

Figure S1. A conceptual framework of Assessment System for Contracted Medical Service Providers


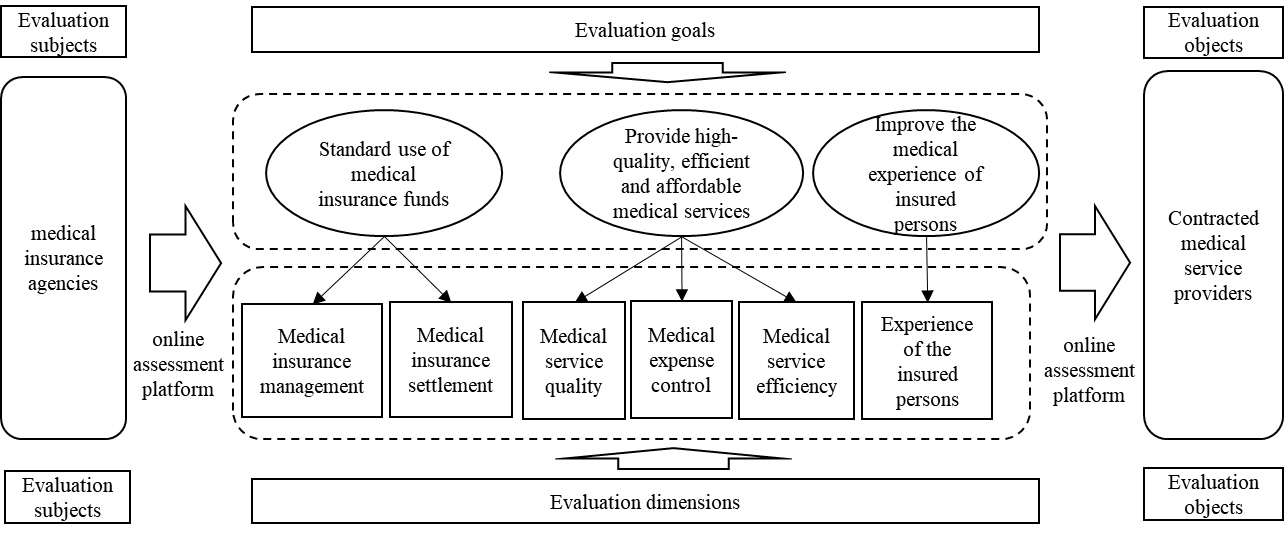

Supplement: Supplementary file 1 [file Data_Sheet_1.docx]
