# Supplementary material for: Development of an assessment tool for designated medical institutions in China——Based on the application of an online assessment system
Source: Front Public Health. 2024 May 6;12:1372821. doi: 10.3389/fpubh.2024.1372821 (PMC11102995; doi:10.3389/fpubh.2024.1372821)
Supplement: Supplementary file 2 [file Data_Sheet_2.docx]

**Appendix 2**

Table S1. Results of two rounds of expert consultation on the proposed indicators

|  | **Round 1** | | | | | **Round 2** | | | | |
| --- | --- | --- | --- | --- | --- | --- | --- | --- | --- | --- |
|  | **Significance** | | **Sensitivity** | | **Outcome** | **Significance** | | **Sensitivity** | | **Outcome** |
|  | 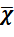 | **CV** | 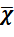 | **CV** |  | 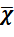 | **CV** | 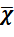 | **CV** |  |
| 1. **Medical insurance management** | 8.00 | 0.11 | 7.85 | 0.11 | Accepted | 8.32 | 0.08 | 8.21 | 0.10 | Accepted |
| **1.1 Basic construction** | 7.75 | 0.12 | 7.70 | 0.12 | Accepted | 8.00 | 0.10 | 8.26 | 0.08 | Accepted |
| 1.1.1 Establish medical insurance department | 8.40 | 0.08 | 8.15 | 0.10 | Accepted | 8.27 | 0.09 | 8.11 | 0.09 | Accepted |
| 1.1.2 Build bylaws and policies | 8.50 | 0.07 | 7.80 | 0.10 | Accepted | 8.27 | 0.15 | 8.16 | 0.09 | Accepted |
| **1.2 Human resource management** | 8.10 | 0.11 | 8.10 | 0.10 | Accepted | 7.91 | 0.11 | 8.42 | 0.08 | Accepted |
| 1.2.1 Records management of physicians | 8.35 | 0.09 | 7.70 | 0.11 | Accepted | 8.43 | 0.07 | 8.26 | 0.10 | Accepted |
| 1.2.2 Records accuracy of physicians | 8.25 | 0.10 | 7.60 | 0.12 | Accepted | 8.13 | 0.07 | 8.26 | 0.08 | Accepted |
| 1.2.3 Insurance settlement personnel | 8.20 | 0.08 | 7.95 | 0.11 | Accepted | 8.02 | 0.08 | 8.16 | 0.09 | Accepted |
| **1.3 Information system** | 7.85 | 0.13 | 7.85 | 0.11 | Accepted | 8.00 | 0.09 | 8.26 | 0.09 | Accepted |
| 1.3.1 Establish medical insurance information management department | 7.80 | 0.11 | 7.65 | 0.10 | Accepted | 7.97 | 0.11 | 8.21 | 0.09 | Accepted |
| 1.3.2 Connect medical insurance network | 8.45 | 0.09 | 8.15 | 0.08 | Accepted | 8.34 | 0.10 | 8.26 | 0.10 | Accepted |
| 1.3.3 Equip auxiliary equipment in computer room | 8.00 | 0.11 | 8.15 | 0.09 | Accepted | 8.07 | 0.10 | 8.05 | 0.08 | Accepted |
| 1.3.4 Equip intelligent monitoring system of basic medical insurance | 8.40 | 0.09 | 8.15 | 0.10 | Accepted | 7.91 | 0.12 | 7.95 | 0.10 | Accepted |
| 1.3.5 Establish doctor (nursing) workstation | 7.95 | 0.11 | 7.85 | 0.10 | Accepted | 8.00 | 0.15 | 8.00 | 0.11 | Accepted |
| 1.3.6 Internet security | 8.35 | 0.10 | 8.00 | 0.10 | Accepted | 8.24 | 0.06 | 7.89 | 0.11 | Accepted |
| 1.3.7 Contingency plan for information system | 8.05 | 0.10 | 7.95 | 0.10 | Accepted | 8.17 | 0.11 | 8.16 | 0.08 | Accepted |
| **1.4 Medical insurance business** | 8.55 | 0.08 | 8.05 | 0.09 | Accepted | 8.43 | 0.08 | 7.95 | 0.11 | Accepted |
| 1.4.1 Sign of designated medical institution | 8.35 | 0.10 | 8.20 | 0.09 | Accepted | 8.34 | 0.11 | 8.47 | 0.08 | Accepted |
| 1.4.2 Monitoring equipment in medical insurance service area | 8.00 | 0.09 | 8.10 | 0.09 | Accepted | 8.06 | 0.10 | 8.11 | 0.11 | Accepted |
| 1.4.3 Medical insurance policy consulting service | 8.05 | 0.09 | 8.05 | 0.09 | Accepted | 8.07 | 0.10 | 7.74 | 0.09 | Accepted |
| 1.4.4 Medical insurance policy training for medical personnel | 8.40 | 0.08 | 8.15 | 0.09 | Accepted | 8.32 | 0.14 | 8.00 | 0.09 | Accepted |
| 1.4.5 Publicity of medical insurance complaint channels | 8.35 | 0.09 | 8.00 | 0.13 | Accepted | 8.18 | 0.09 | 8.26 | 0.08 | Accepted |
| 1.4.10 Implementation of additional agreements of specific institutions | 8.60 | 0.06 | 7.65 | 0.10 | Accepted | 8.44 | 0.05 | 7.53 | 0.10 | Accepted |
| **1.5 Drug procurement** | 7.65 | 0.14 | 7.65 | 0.12 | Accepted | 8.38 | 0.09 | 8.16 | 0.09 | Accepted |
| 1.5.1 Purchase, sales and deposit record | 7.40 | 0.08 | 7.60 | 0.10 | Accepted | 7.62 | 0.10 | 7.42 | 0.09 | Accepted |
| 1.5.2 Application of national procurement platform | 7.85 | 0.13 | 7.75 | 0.11 | Accepted | 8.54 | 0.09 | 8.00 | 0.10 | Accepted |
| 1.5.4 Completely product authorization information | 7.75 | 0.09 | 7.05 | 0.16 | Accepted | 7.66 | 0.09 | 7.79 | 0.09 | Accepted |
| 1.5.5 Proportion of centralized procurement drugs | 7.55 | 0.09 | 7.60 | 0.13 | Accepted | 7.69 | 0.10 | 7.79 | 0.09 | Accepted |
| 1. **Medical insurance settlement** | 8.45 | 0.08 | 8.10 | 0.12 | Accepted | 8.16 | 0.10 | 7.95 | 0.10 | Accepted |
| **2.1 Claims settlement requirement** | 8.35 | 0.10 | 8.00 | 0.11 | Accepted | 8.22 | 0.12 | 8.05 | 0.12 | Accepted |
| 2.1.1 Claims settlement materials | 8.40 | 0.09 | 7.35 | 0.12 | Accepted | 8.19 | 0.18 | 7.58 | 0.10 | Accepted |
| 2.1.2 Scope of claim settlement | 8.15 | 0.10 | 7.60 | 0.11 | Accepted | 8.09 | 0.19 | 7.58 | 0.11 | Accepted |
| 2.1.3 Issue settlement bills | 7.90 | 0.12 | 8.05 | 0.10 | Accepted | 7.87 | 0.09 | 7.63 | 0.14 | Accepted |
| 2.1.4 Settlement of agreed diagnosis and treatment items | 8.55 | 0.07 | 7.65 | 0.11 | Accepted | 8.32 | 0.11 | 7.89 | 0.12 | Accepted |
| 2.1.5 Accidental injury settlement | 6.40 | 0.14 | 6.65 | 0.14 | Deleted |  |  |  |  |  |
| 2.1.6 Cross regional medical insurance settlement | 6.90 | 0.11 | 7.35 | 0.12 | Deleted |  |  |  |  |  |
| 2.1.7 Medical dispute settlement | 6.25 | 0.16 | 6.75 | 0.11 | Deleted |  |  |  |  |  |
| **2.2 Reconciliation management** | 8.65 | 0.06 | 8.20 | 0.08 | Accepted | 8.23 | 0.10 | 8.37 | 0.07 | Accepted |
| 2.2.1 Overdue days of reconciliation | 8.30 | 0.08 | 8.10 | 0.08 | Accepted | 8.26 | 0.15 | 8.32 | 0.08 | Accepted |
| 2.2.2 Proportion of daily reconciliation deduction amount | 8.20 | 0.08 | 8.10 | 0.08 | Accepted | 8.04 | 0.12 | 8.32 | 0.15 | Accepted |
| 1. **Medical service quality** | 8.60 | 0.07 | 8.05 | 0.09 | Accepted | 8.10 | 0.09 | 7.79 | 0.12 | Accepted |
| **3.1 Medical service management** | 8.20 | 0.10 | 7.80 | 0.09 | Accepted | 8.07 | 0.09 | 7.95 | 0.10 | Accepted |
| 3.1.1 Identify the insured correctly | 8.25 | 0.10 | 7.55 | 0.12 | Accepted | 8.24 | 0.11 | 7.58 | 0.17 | Accepted |
| 3.1.2 Qualified medical record | 8.35 | 0.09 | 7.25 | 0.13 | Accepted | 8.4 | 0.10 | 7.53 | 0.16 | Accepted |
| 3.1.3 Medical expense inquiry service | 8.40 | 0.09 | 8.10 | 0.09 | Accepted | 8.44 | 0.07 | 8.42 | 0.11 | Accepted |
| 3.1.4 Registration and filing of external inspection and treatment | 7.60 | 0.07 | 7.55 | 0.12 | Accepted | 7.92 | 0.07 | 8.11 | 0.11 | Accepted |
| 3.1.5 Standard use of family sickbeds | 8.05 | 0.09 | 7.40 | 0.10 | Accepted | 8.11 | 0.08 | 7.42 | 0.12 | Accepted |
| 3.1.6 Outpatient major disease standard certification rate | 7.55 | 0.08 | 7.65 | 0.11 | Accepted | 7.69 | 0.10 | 6.60 | 0.15 | Deleted |
| 3.1.7 Discharge and admission management | 7.95 | 0.14 | 7.45 | 0.15 | Accepted | 8.13 | 0.08 | 6.25 | 0.13 | Deleted |
| 3.1.8 Outpatient prescription outsourcing service | 7.60 | 0.17 | 7.20 | 0.14 | Accepted | 7.91 | 0.09 | 8.05 | 0.14 | Accepted |
| 3.1.9 Dispensing management | 6.65 | 0.16 | 6.95 | 0.14 | Deleted |  |  |  |  |  |
| 3.1.10 Remote medical services | 6.80 | 0.15 | 7.30 | 0.09 | Deleted |  |  |  |  |  |
| 3.1.11 Hospitals reject patients without justifiable reasons | 8.45 | 0.08 | 8.15 | 0.10 | Accepted | 8.22 | 0.10 | 8.16 | 0.08 | Accepted |
| 3.1.12 Scoring of bad practice of medical institutions | 8.45 | 0.06 | 8.25 | 0.09 | Accepted | 8.41 | 0.09 | 8.11 | 0.14 | Accepted |
| **3.2 Health care quality management** | 8.55 | 0.08 | 7.75 | 0.11 | Accepted | 8.38 | 0.06 | 7.74 | 0.08 | Accepted |
| 3.2.1 Qualified rate of inspection | 8.40 | 0.11 | 7.45 | 0.12 | Accepted | 8.43 | 0.12 | 8.05 | 0.07 | Accepted |
| 3.2.2 Proportion of default amount of drugs with payment limitation | 8.30 | 0.09 | 8.25 | 0.08 | Accepted | 8.18 | 0.10 | 7.84 | 0.10 | Accepted |
| 3.2.3 Discharge medication variety and diagnostic compliance rate | 7.85 | 0.09 | 7.05 | 0.16 | Accepted | 8.13 | 0.07 | 6.40 | 0.11 | Deleted |
| 3.2.4 Mortality of cases in low-risk group | 7.60 | 0.10 | 7.25 | 0.13 | Accepted | 8.23 | 0.10 | 7.26 | 0.10 | Accepted |
| **4. Medical service efficiency** | 7.95 | 0.12 | 7.90 | 0.10 | Accepted | 8.37 | 0.08 | 8.32 | 0.07 | Accepted |
| **4.1 Convenient medical treatment** | 8.10 | 0.13 | 8.05 | 0.09 | Accepted | 8.05 | 0.10 | 7.68 | 0.08 | Accepted |
| 4.1.1 Average waiting time after appointment | 8.15 | 0.11 | 7.70 | 0.10 | Accepted | 8.25 | 0.07 | 8.15 | 0.12 | Accepted |
| 4.1.2 Convenience Services and Facilities | 8.30 | 0.07 | 8.25 | 0.10 | Accepted | 7.81 | 0.10 | 8.05 | 0.09 | Accepted |
| **4.2** **Efficient diagnosis and treatment** | 8.50 | 0.14 | 8.00 | 0.15 | Accepted | 8.29 | 0.10 | 7.95 | 0.09 | Accepted |
| 4.2.1 Outpatient return visit rate | 8.20 | 0.09 | 7.95 | 0.10 | Accepted | 8.16 | 0.06 | 7.84 | 0.08 | Accepted |
| 4.2.2 Re admission rate within 15 days after discharge | 8.35 | 0.10 | 7.80 | 0.13 | Accepted | 8.20 | 0.08 | 7.74 | 0.08 | Accepted |
| 4.2.5 Inpatient outpatient ratio | 8.05 | 0.12 | 7.60 | 0.10 | Accepted | 7.90 | 0.10 | 7.53 | 0.15 | Accepted |
| 4.2.6 Mutual recognition and sharing of inspection results | 6.75 | 0.15 | 7.40 | 0.12 | Deleted |  |  |  |  |  |
| **5. Medical expense** | 8.50 | 0.08 | 8.20 | 0.09 | Accepted | 8.37 | 0.09 | 7.95 | 0.11 | Accepted |
| **5.1 Growth rate of medical expenses** | 8.25 | 0.09 | 7.90 | 0.12 | Accepted | 8.02 | 0.14 | 7.95 | 0.08 | Accepted |
| 5.1.1 Proportion of medical service income | 8.00 | 0.07 | 7.95 | 0.10 | Accepted | 7.85 | 0.10 | 8.21 | 0.10 | Accepted |
| 5.1.2 Increase in average outpatient cost per time | 8.45 | 0.08 | 8.00 | 0.09 | Accepted | 8.23 | 0.13 | 8.37 | 0.08 | Accepted |
| 5.1.3 Increase in average hospitalization cost per time | 8.15 | 0.10 | 8.10 | 0.11 | Accepted | 8.30 | 0.10 | 8.37 | 0.08 | Accepted |
| 5.1.4 Increase in average drug cost per outpatient | 7.50 | 0.11 | 7.95 | 0.10 | Accepted | 7.70 | 0.10 | 7.95 | 0.11 | Accepted |
| 5.1.5 Increase in average drug cost per hospitalization | 7.65 | 0.09 | 8.00 | 0.09 | Accepted | 7.70 | 0.12 | 7.95 | 0.10 | Accepted |
| **5.2 Reasonable medical charges** | 8.50 | 0.08 | 7.85 | 0.18 | Accepted | 8.17 | 0.10 | 7.74 | 0.12 | Accepted |
| 5.2.5 Cost shifting of exceeding medical insurance settlement | 8.45 | 0.09 | 7.50 | 0.11 | Accepted | 8.13 | 0.11 | 7.58 | 0.09 | Accepted |
| 5.2.6 Implementation of copay rate of medical insurance | 8.70 | 0.08 | 7.50 | 0.11 | Accepted | 8.50 | 0.09 | 7.74 | 0.19 | Accepted |
| 5.2.7 Self payment rate | 6.60 | 0.14 | 7.50 | 0.11 | Deleted |  |  |  |  |  |
| 5.2.9 Standardizing charge for newly increased medical service | 8.10 | 0.08 | 7.70 | 0.12 | Accepted | 8.29 | 0.14 | 7.63 | 0.18 | Accepted |
| **6. Experience of the insured** | 8.20 | 0.08 | 8.00 | 0.09 | Accepted | 8.32 | 0.07 | 8.16 | 0.09 | Accepted |
| **6.1 The insured’s rights** | 8.45 | 0.07 | 7.95 | 0.14 | Accepted | 8.39 | 0.08 | 7.63 | 0.11 | Accepted |
| 6.1.1 Signing of informed consent | 8.50 | 0.08 | 7.60 | 0.11 | Accepted | 8.36 | 0.13 | 7.79 | 0.11 | Accepted |
| 6.1.2 Information security | 8.75 | 0.06 | 7.60 | 0.12 | Accepted | 8.17 | 0.07 | 8.16 | 0.09 | Accepted |
| **6.2 Evaluation of the insured** | 8.65 | 0.07 | 7.90 | 0.10 | Accepted | 8.33 | 0.10 | 7.63 | 0.09 | Accepted |
| 6.2.1 Subjective satisfaction of the insured | 8.45 | 0.06 | 8.15 | 0.09 | Accepted | 8.11 | 0.08 | 7.58 | 0.08 | Accepted |
| 6.2.2 Complaints of the insured | 8.50 | 0.07 | 8.20 | 0.09 | Accepted | 8.31 | 0.08 | 8.21 | 0.08 | Accepted |
