# Supplementary material for: Development of an assessment tool for designated medical institutions in China——Based on the application of an online assessment system
Source: Front Public Health. 2024 May 6;12:1372821. doi: 10.3389/fpubh.2024.1372821 (PMC11102995; doi:10.3389/fpubh.2024.1372821)
Supplement: Supplementary file 3 [file Data_Sheet_3.docx]

**Appendix 3**

Table S2. Assessment Index System for DMIs

| **Indicators** | **Indicator description** | **Data sources** | **Indicator direction** |
| --- | --- | --- | --- |
| **1. Medical insurance management** |  |  |  |
| **1.1 Basic construction** |  |  |  |
| 1.1.1 Establish medical insurance department | Set up a medical insurance office | Hospital self report | Positive directional |
| 1.1.2 Build bylaws and policies | establish the regulations required by basic medical insurance | Hospital self report | Positive directional |
| **1.2 Human resource management** |  |  |  |
| 1.2.1 Records management of physicians | Physicians’ record rate=Number of physicians in hospital’s database/ Actual number of physician in hospital | Records of information department of medical insurance agency, Hospital self report | Positive directional |
| 1.2.2 Records accuracy of physicians | Accuracy rate=Number of correct records of physicians in database/Number of total records | Records of information department of medical insurance agency, Hospital self report | Positive directional |
| 1.2.3 Insurance settlement personnel | Assign an expertise to be responsible for the review of medical insurance settlement expenses and daily reconciliation work | Hospital self report | Positive directional |
| **1.3 Information system** |  |  |  |
| 1.3.1 Establish medical insurance information management department | Set up a department or personnel responsible for the management of medical insurance information system | Hospital self report | Positive directional |
| 1.3.2 Connect medical insurance network | Equip facilities and connect the medical insurance network | Hospital self report | Positive directional |
| 1.3.3 Equip auxiliary equipment in computer room | Equip UPS power and temperature control in computer room | Hospital self report | Positive directional |
| 1.3.4 Equip intelligent monitoring system of basic medical insurance | Embed medical insurance intelligent monitoring rules in hospital’s information system | Records of information department of medical insurance agency | Positive directional |
| 1.3.5 Establish doctor (nursing) workstation | Establish doctor (nursing) workstation | Hospital self report | Positive directional |
| 1.3.6 Internet security | The network is physically or logically isolated from the Internet | Records of information department of medical insurance agency | Positive directional |
| 1.3.7 Contingency plan for information system | Establish information system contingency plan and organize drills | Hospital self report | Positive directional |
| **1.4 Medical insurance business** |  |  |  |
| 1.4.1 Sign of designated medical institution | Hung the signboard of designated medical institution at a conspicuous place at the main entrance and exit | Hospital self report | Positive directional |
| 1.4.2 Monitoring equipment in medical insurance service area | Equip monitoring equipment in medical insurance service area, and ensure the monitoring video is clear | Hospital self report | Positive directional |
| 1.4.3 Medical insurance policy consulting service | Provide medical insurance policy consulting services to the insured | Hospital self report | Positive directional |
| 1.4.4 Medical insurance policy training for medical personnel | Conduct medical insurance policy training for medical personnel | Hospital self report | Positive directional |
| 1.4.5 Publicity of medical insurance complaint channels | Publicize the telephone number of medical insurance complaint | Hospital self report | Positive directional |
| 1.4.10 Implementation of additional agreements of specific institutions | Implementation of additional agreements of specific institutions | Records of medical insurance agency | Positive directional |
| **1.5 Drug procurement** |  |  |  |
| 1.5.1 Purchase, sales and deposit record | Record rate of purchase, sales and deposit of drugs and medical devices | Hospital self report | Positive directional |
| 1.5.2 Application of national procurement platform | Connect the hospital management information system with the national procurement platform | Records of pharmaceutical centralized bidding procurement affairs management office | Positive directional |
| 1.5.4 Completely product authorization information | The medical devices purchased by the hospital have complete product authorization information | Records of pharmaceutical centralized bidding procurement affairs management office | Positive directional |
| 1.5.5 Proportion of centralized procurement drugs | Proportion of centralized procurement drugs =Purchase amount of bid winning drugs/ Purchase amount of the same kind of drugs | Records of Health Commission, hospital self report | Positive directional |
| **2. Medical insurance settlement** |  |  |  |
| **2.1 Claims settlement requirement** |  |  |  |
| 2.1.1 Claims settlement materials | Medical expense reimbursement materials are complete | Audit division of medical insurance agency | Positive directional |
| 2.1.2 Scope of claim settlement | Medical services declared for reimbursement should be approved by the health administrative department | Audit division of medical insurance agency | Positive directional |
| 2.1.3 Issue settlement bills | Issue special invoices for medical expense settlement | Hospital self report | Positive directional |
| 2.1.4 Settlement of agreed diagnosis and treatment items | Diagnosis and treatment items for medical insurance settlement have been approved | Audit division of medical insurance agency | Positive directional |
| **2.2 Reconciliation management** |  |  |  |
| 2.2.1 Overdue days of reconciliation | Overdue days of annual daily reconciliation | Audit division of medical insurance agency | Negative directional |
| 2.2.2 Proportion of daily reconciliation deduction amount | Proportion of daily reconciliation deduction amount=Amount of annual daily reconciliation deduction expense/ Total amount of annual medical insurance settlement | Audit division of medical insurance agency | Negative directional |
| **3. Medical service quality** |  |  |  |
| **3.1 Medical service management** |  |  |  |
| 3.1.1 Identify the insured correctly | Check the identification of the insured | Hospital self report | Positive directional |
| 3.1.2 Qualified medical record | Establish medical records for the insured based on the uniform standard | Records of Health Commission | Positive directional |
| 3.1.3 Medical expense inquiry service | The hospital provides medical expense inquiry service and medical expense list | Hospital self report | Positive directional |
| 3.1.4 Registration and filing of external inspection and treatment | Registration and filing rate of external inspection and external treatment= number of registration and filing of external inspection and treatment /the total number of external inspection and treatment | Fund department of medical Insurance agency、hospital self report | Positive directional |
| 3.1.5 Standard use of family sickbeds | Provide family sickbed service according to the standard | Records of Health Commission | Positive directional |
| 3.1.8 Outpatient prescription outsourcing service | The outpatient prescription should be stamped with the doctor's seal | Hospital self report | Positive directional |
| 3.1.11 Hospitals reject patients without justifiable reasons | Complaint rate of Hospitals’ reject patients events= Number of patients rejected by the hospital/Total number of hospital visits | Records of Medical insurance complaint hotline | Negative directional |
| 3.1.12 Scoring of bad practice of medical institutions | Scoring of bad practice of medical institutions | Records of Health Commission | Negative directional |
| **3.2 Health care quality management** |  |  |  |
| 3.2.1 Qualified rate of inspection | Qualification rate of inspection= Number of spot-checked qualified inspection/total number of spot-checked inspection | Records of Health Commission | Positive directional |
| 3.2.2 Proportion of default amount of drugs with payment limitation | Proportion of default amount of drugs with payment limitation= Default amount of drugs with payment limitation/ Total amount of drugs with payment limitation | Audit division of medical insurance agency | Negative directional |
| 3.2.4 Mortality of cases in low-risk group | Mortality of cases in low-risk group=Number of deaths in low-risk group/Total number of patients in low-risk group | Records of medical insurance agency | Negative directional |
| **4. Medical service efficiency** |  |  |  |
| **4.1 Convenient medical treatment** |  |  |  |
| 4.1.1 Average waiting time after appointment | Average waiting time of outpatient after appointment (minutes) | Hospital self report | Negative directional |
| 4.1.2 Convenience Services and Facilities | Provide convenience services and facilities such as accessible facilities and auxiliary wheelchairs for the disabled, suitable parking areas and other facilities or services | Hospital self report | Positive directional |
| **4.2** **Efficient diagnosis and treatment** |  |  |  |
| 4.2.1 Outpatient return visit rate | Outpatient return visit rate=Number of Outpatient return visits of the same disease/ Number of outpatients | Hospital self report | Negative directional |
| 4.2.2 Re admission rate within 15 days after discharge | Re admission rate within 15 days after discharge=Number of re-inpatients within 15 days after discharge/ Total number of discharged patients | Hospital self report | Negative directional |
| 4.2.5 Inpatient outpatient ratio | Inpatient outpatient ratio=Number of inpatients/Number of outpatients | Hospital self report | Negative directional |
| **5. Medical expense** |  |  |  |
| **5.1 Growth rate of medical expenses** |  |  |  |
| 5.1.1 Proportion of medical service income | Proportion of medical service income=medical service income/medical income | Hospital self report | Positive directional |
| 5.1.2 Increase in average outpatient cost per time | Increase in average outpatient cost per time= (average outpatient cost per time in the year/ average outpatient cost in last year)-1 | Hospital self report | Negative directional |
| 5.1.3 Increase in average hospitalization cost per time | Increase in average hospitalization cost per time= (average hospitalization cost per time in the year/ average hospitalization cost in last year)-1 | Hospital self report | Negative directional |
| 5.1.4 Increase in average drug cost per outpatient | Increase in average drug cost per outpatient = (average drug cost per outpatient in the year/ average drug cost per outpatient in last year)-1 | Hospital self report | Negative directional |
| 5.1.5 Increase in average drug cost per hospitalization | Increase in average drug cost per hospitalization = (average drug cost per hospitalization in the year/ average drug cost per hospitalization in last year)-1 | Hospital self report | Negative directional |
| **5.2 Reasonable medical charges** |  |  |  |
| 5.2.5 Cost shifting of exceeding medical insurance settlement | The hospital shall not transfer the medical expenses beyond target control to the insured at their own expense | Medical insurance supervision institute | Negative directional |
| 5.2.6 Implementation of copay rate of medical insurance | The designated medical institutions shall strictly implement the copay rate of medical insurance | Audit division of medical insurance agency | Negative directional |
| 5.2.9 Standardizing charge for newly increased medical service | Newly increased medical service can charge after approval | Audit division of medical insurance agency | Positive directional |
| **6.** **Experience of the insured** |  |  |  |
| **6.1 The insured’s rights** |  |  |  |
| 6.1.1 Signing of informed consent | The informed consent of the insured or their families should be sighed in advance when they were provided self-funded service | Hospital self report | Positive directional |
| 6.1.2 Information security | The hospital shall not disclose the medical information of the insured at will | Hospital self report | Positive directional |
| **6.2 Evaluation of the insured** |  |  |  |
| 6.2.1 Subjective satisfaction of the insured | The insured gives a subjective score of designated medical institutions | Records of Health Commission | Positive directional |
| 6.2.2 Complaints of the insured | The number of verified complaints by the insured on the medical insurance complaint hotline | Records of Medical insurance complaint hotline | Negative directional |
