# Supplementary material for: Development of an assessment tool for designated medical institutions in China——Based on the application of an online assessment system
Source: Front Public Health. 2024 May 6;12:1372821. doi: 10.3389/fpubh.2024.1372821 (PMC11102995; doi:10.3389/fpubh.2024.1372821)
Supplement: Supplementary file 4 [file Data_Sheet_4.docx]

**Appendix 4**

Table S3. Dimensionless results of indicators according to the data type

| **Indicators** | **Dimensionless method** | **Assignment criterion** |
| --- | --- | --- |
| **1. Medical insurance management** |  |  |
| **1.1 Basic construction** |  |  |
| 1.1.1 Establish medical insurance department | Multi-condition assignment | Assignment based on the completion of each condition. For example, assign 0.5 point for department establishment, and assign 0.5 point for manning with full-time staff. |
| 1.1.2 Build bylaws and policies | Proportional assignment | Assign values proportionally based on the completion degree. |
| **1.2 Human resource management** |  |  |
| 1.2.1 Records management of physicians | Proportional assignment | Assign values proportionally based on the completion degree. |
| 1.2.2 Records accuracy of physicians | Proportional assignment | Assign values proportionally based on the completion degree. |
| 1.2.3 Insurance settlement personnel | Multi-condition assignment | Assignment based on the completion of each condition. Assign 0.5 point for staffing and 0.5 point for the personnel satisfying work requirements. |
| **1.3 Information system** |  |  |
| 1.3.1 Establish medical insurance information management department | "0-1" assignment | Assign a value of 1 if the requirement is met and assign 0 if not. |
| 1.3.2 Connect medical insurance network | "0-1" assignment | Assign a value of 1 if the requirement is met and assign 0 if not. |
| 1.3.3 Equip auxiliary equipment in computer room | "0-1" assignment | Assign a value of 1 if the requirement is met and assign 0 if not. |
| 1.3.4 Equip intelligent monitoring system of basic medical insurance | "0-1" assignment | Assign a value of 1 if the requirement is met and assign 0 if not. |
| 1.3.5 Establish doctor (nursing) workstation | "0-1" assignment | Assign a value of 1 if the requirement is met and assign 0 if not. |
| 1.3.6 Internet security | "0-1" assignment | Assign 0 if the institution was found to have internal users connecting to the external network without permission. |
| 1.3.7 Contingency plan for information system | Multi-condition assignment | Assignment based on the completion of each condition. Assign 0.5 point for establishing contingency plan and 0.5 point for organizing drills. |
| **1.4 Medical insurance business** |  |  |
| 1.4.1 Sign of designated medical institution | "0-1" assignment | Assign a value of 1 if the requirement is met, and assign 0 if not |
| 1.4.2 Monitoring equipment in medical insurance service area | Multi-condition assignment | Assignment based on the completion of each condition. Assign 0.5 point for equipped with monitoring equipment and 0.5 point for clear monitoring video. |
| 1.4.3 Medical insurance policy consulting service | "0-1" assignment | Assign a value of 1 if the requirement is met and assign 0 if not. |
| 1.4.4 Medical insurance policy training for medical personnel | "0-1" assignment | Assign a value of 1 if the requirement is met and assign 0 if not. |
| 1.4.5 Publicity of medical insurance complaint channels | "0-1" assignment | Assign a value of 1 if the requirement is met and assign 0 if not. |
| 1.4.10 Implementation of additional agreements of specific institutions | "0-1" assignment | Assign a value of 1 if the requirement is met and assign 0 if not. |
| **1.5 Drug procurement** |  |  |
| 1.5.1 Purchase, sales and deposit record | Proportional assignment | Assign values proportionally based on the completion degree. |
| 1.5.2 Application of national procurement platform | "0-1" assignment | Assign a value of 1 if the requirement is met and assign 0 if not. |
| 1.5.4 Completely product authorization information | "0-1" assignment | Assign a value of 1 if the requirement is met and assign 0 if not. |
| 1.5.5 Proportion of centralized procurement drugs | Horizontal comparison assignment | Assign 1 if the value higher than or equal to the average of DMIs at the same level, otherwise, assign $y_{i}=\frac{X_{i}}{\bar{X}}$. |
| **2. Medical insurance settlement** |  |  |
| **2.1 Claims settlement requirement** |  |  |
| 2.1.1 Claims settlement materials | "0-1" assignment | Assign a value of 1 if the requirement is met and assign 0 if not. |
| 2.1.2 Scope of claim settlement | "0-1" assignment | Assign a value of 1 if the requirement is met and assign 0 if not. |
| 2.1.3 Issue settlement bills | "0-1" assignment | Assign a value of 1 if the requirement is met and assign 0 if not. |
| 2.1.4 Settlement of agreed diagnosis and treatment items | "0-1" assignment | Assign a value of 1 if the requirement is met and assign 0 if not. |
| **2.2 Reconciliation management** |  |  |
| 2.2.1 Overdue days of reconciliation | Min-max normalization | Assign the value according to the formula $y_{i}=\frac{\max\left（ x_{i} \right）-x_{i}}{\max\left（ x_{i} \right）-min（x_{i}）}$. |
| 2.2.2 Proportion of daily reconciliation deduction amount | Horizontal comparison assignment | Assign 1 if the value lower than or equal to the average of DMIs at the same level, otherwise, assign$y_{i}=\frac{\bar{X}}{X_{i}}$. |
| **3. Medical service quality** |  |  |
| **3.1 Medical service management** |  |  |
| 3.1.1 Identify the insured correctly | "0-1" assignment | Assign a value of 1 if the requirement is met and assign 0 if not. |
| 3.1.2 Qualified medical record | Proportional assignment | Assign values proportionally based on the completion degree. |
| 3.1.3 Medical expense inquiry service | "0-1" assignment | Assign a value of 1 if the requirement is met and assign 0 if not. |
| 3.1.4 Registration and filing of external inspection and treatment | Proportional assignment | Assign values proportionally based on the completion degree. |
| 3.1.5 Standard use of family sickbeds | Proportional assignment | Assign values proportionally based on the completion degree. |
| 3.1.8 Outpatient prescription outsourcing service | "0-1" assignment | Assign a value of 1 if the requirement is met and assign 0 if not. |
| 3.1.11 Hospitals reject patients without justifiable reasons | Proportional assignment | Assign values proportionally based on the completion degree. |
| 3.1.12 Scoring of bad practice of medical institutions | Segment assignment | Assign 0 if the score of bad practice of medical institutions more than 10 points, and assign proportionally if below 10 points. |
| **3.2 Health care quality management** |  |  |
| 3.2.1 Qualified rate of inspection | Proportional assignment | Assign values proportionally based on the completion degree. |
| 3.2.2 Proportion of default amount of drugs with payment limitation | Horizontal comparison assignment | Assign 1 if the value lower than or equal to the average of DMIs at the same level, otherwise, assign$y_{i}=\frac{\bar{X}}{X_{i}}$. |
| 3.2.4 Mortality of cases in low-risk group | Horizontal comparison assignment | Assign 1 if the value lower than or equal to the average of DMIs at the same level, otherwise, assign$y_{i}=\frac{\bar{X}}{X_{i}}$. |
| **4. Medical service efficiency** |  |  |
| **4.1 Convenient medical treatment** |  |  |
| 4.1.1 Average waiting time after appointment | Horizontal comparison assignment | Assign 1 if the value lower than or equal to the average of DMIs at the same level, otherwise, assign$y_{i}=\frac{\bar{X}}{X_{i}}$. |
| 4.1.2 Convenience Services and Facilities | "0-1" assignment | Assign a value of 1 if the requirement is met and assign 0 if not. |
| **4.2** **Efficient diagnosis and treatment** |  |  |
| 4.2.1 Outpatient return visit rate | Horizontal comparison assignment | Assign 1 if the value lower than or equal to the average of DMIs at the same level, otherwise, assign$y_{i}=\frac{\bar{X}}{X_{i}}$. |
| 4.2.2 Re admission rate within 15 days after discharge | Horizontal comparison assignment | Assign 1 if the value lower than or equal to the average of DMIs at the same level, otherwise, assign$y_{i}=\frac{\bar{X}}{X_{i}}$. |
| 4.2.5 Inpatient outpatient ratio | Horizontal comparison assignment | Assign 1 if the value lower than or equal to the average of DMIs at the same level, otherwise, assign$y_{i}=\frac{\bar{X}}{X_{i}}$. |
| **5. Medical expense** |  |  |
| **5.1 Growth rate of medical expenses** |  |  |
| 5.1.1 Proportion of medical service income | Segment assignment | Assign 1 if the value higher than or equal to 50%, otherwise assign proportionally. |
| 5.1.2 Increase in average outpatient cost per time | Horizontal comparison assignment | Assign 1 if the value lower than or equal to the average of DMIs at the same level, otherwise, assign$y_{i}=\frac{\bar{X}}{X_{i}}$. |
| 5.1.3 Increase in average hospitalization cost per time | Horizontal comparison assignment | Assign 1 if the value lower than or equal to the average of DMIs at the same level, otherwise, assign$y_{i}=\frac{\bar{X}}{X_{i}}$. |
| 5.1.4 Increase in average drug cost per outpatient | Horizontal comparison assignment | Assign 1 if the value lower than or equal to the average of DMIs at the same level, otherwise, assign$y_{i}=\frac{\bar{X}}{X_{i}}$. |
| 5.1.5 Increase in average drug cost per hospitalization | Horizontal comparison assignment | Assign 1 if the value lower than or equal to the average of DMIs at the same level, otherwise, assign$y_{i}=\frac{\bar{X}}{X_{i}}$. |
| **5.2 Reasonable medical charges** |  |  |
| 5.2.5 Cost shifting of exceeding health insurance settlement | "0-1" assignment | Assign 0 once the DMI is found to have cost shifting of exceeding health insurance settlement according to the insurance settlement records. |
| 5.2.6 Implementation of copay rate of medical insurance | Horizontal comparison assignment | Assign 1 if the value lower than or equal to the average of DMIs at the same level, otherwise, assign$y_{i}=\frac{\bar{X}}{X_{i}}$. |
| 5.2.9 Standardizing charge for newly increased medical service | "0-1" assignment | Assign a value of 1 if the requirement is met and assign 0 if not. |
| **6. Experience of the insured** |  |  |
| **6.1 The insured’s rights** |  |  |
| 6.1.1 Signing of informed consent | "0-1" assignment | Assign 0 once the informed consent forms in the spot check is found to be unqualified. |
| 6.1.2 Information security | "0-1" assignment | Assign a value of 1 if the requirement is met and assign 0 if not. |
| **6.2 Evaluation of the insured** |  |  |
| 6.2.1 Subjective satisfaction of the insured | Segment assignment | Assign 0 if the satisfaction score lower than 60 points, assign 1 if higher than 95, and assign proportionally otherwise. |
| 6.2.2 Complaints of the insured | Min-max normalization | Assign the value according to the formula $y_{i}=\frac{\max\left（ x_{i} \right）-x_{i}}{\max\left（ x_{i} \right）-min（x_{i}）}$. |
